# Carbon ion beam combined with cisplatin effectively disrupts triple negative breast cancer stem-like cells *in vitro*

**DOI:** 10.1186/s12943-015-0429-7

**Published:** 2015-09-04

**Authors:** Sei Sai, Guillaume Vares, Eun Ho Kim, Kumiko Karasawa, Bing Wang, Mitsuru Nenoi, Yoshiya Horimoto, Mitsuhiro Hayashi

**Affiliations:** Medical Physics Research Program, Research Center for Charged Particle Therapy, National Institute of Radiological Sciences, 4-9-1 Anagawa Inage-ku, Chiba, Chiba, 263-8555 Japan; Radiation Risk Reduction Research Program, Research Center for Radiation Protection, National Institute of Radiological Sciences, Chiba, Japan; Division of Heavy Ion Clinical Research, Korea Institute of Radiological and Medical Sciences, 215-4 Gongneung-dong, Nowon-Gu, Seoul, 139-706 South Korea; Research Center Hospital for Charged Particle Therapy, National Institute of Radiological Sciences, Chiba, Japan; Department of Breast Oncology, Juntendo University School of Medicine, Tokyo, Japan; Department of Breast Oncology, Tokyo Medical University Hachioji Medical Center, Tokyo, Japan

**Keywords:** Heavy-ion radiation, Breast cancer stem cell, Cisplatin

## Abstract

**Aims:**

Although a relatively small proportion of all breast cancer (BC), triple negative (TN) BC is responsible for a relatively large proportion of BC deaths because of its worse clinical outcome. To investigate whether a carbon ion beam alone or in combination with cisplatin (CDDP) has a beneficial effect compared to X-rays, we target triple negative (TN) breast cancer stem-like cells (CSCs).

**Methods:**

Human breast CSCs sorted from MDA-MB-231 and MDA-MB-453 cells were treated with a carbon ion beam or X-ray irradiation alone or in combination with CDDP, and then colony, spheroid and tumor formation assays, RT-PCR Array analysis, and immunofluorescence γH2AX foci assay were performed.

**Results:**

The colony, spheroid formation, and tumorigenicity assays confirmed that CD44+/CD24- and ESA+/CD24- cells have CSC properties in MDA-MB-231 and MDA-MB-453 cells, respectively. The proportion of CSCs was more enriched after CDDP combination with either X-ray or carbon ion beam, however carbon ion beam combined with CDDP significantly suppressed colony and spheroid formation and more significantly inhibited cell cycle progression (sub-G1 arrest) compared to X-ray combined with CDDP or carbon ion beam alone. RT-PCR Array analysis showed that carbon ion beam combined with CDDP significantly induced apoptosis-related Cytochrome c, almost completely eliminated expression of the CSC markers CD44 and ESA, and significantly inhibited angiogenesis, and metastasis-related HIF1α and CD26 compared to carbon ion beam alone, X-ray alone, or X-ray combined with CDDP. The immunofluorescence assay showed that not only the number but also the size of γH2AX foci in CSCs were larger 24 h after carbon ion beam combined with CDDP compared to those of X-ray alone and X-ray combined with CDDP.

**Conclusions:**

Carbon ion beam combined with CDDP has superior potential to kill TN breast CSCs with irreparable severe DNA damage and enhanced apoptosis.

**Electronic supplementary material:**

The online version of this article (doi:10.1186/s12943-015-0429-7) contains supplementary material, which is available to authorized users.

## Introduction

Human breast cancer (BC) has become one of the leading causes of cancer-related death for women worldwide, and it is rapidly increasing in Asian countries including Japan [1–3]. BC represents a group of highly heterogeneous lesions consisting of morphologically distinct subtypes [4], with different molecular and biochemical signatures [5].

Triple-negative breast cancers (TNBC), defined as tumors that are negative for estrogen receptor (ER), progesterone receptor (PR) and human epidermal growth factor receptor 2 (HER2), nowadays represent the focus of increasing interest at the clinical, biological and epidemiological level [6–8], due to the aggressive behavior of the tumor, poor prognosis and present lack of targeted therapies [9–11]. According to current estimates, TNBC accounts for 10–17 % of all BC, depending on thresholds used to define ER and PR positivity and HER2 overexpression [12, 13]. Despite its relatively small proportion among all BCs, TNBC is responsible for a relatively large proportion of BC deaths, due to its generally aggressive clinical course.

The very high rate of heterogeneity in BC cell phenotypes [14], accompanied by the dynamic plasticity of the BC microenvironment [15], make tumor categorization a demanding task, especially in relation to therapeutic responses and risk of disease progression [16]. Breast cancer stem-like cell (BCSC) populations have recently been identified based on the cell membrane markers CD44+/CD24-/ ESA+ cells [17, 18]. CSCs represent the tumor’s subpopulation with the highest capacity to drive its growth, invasion and metastasis. BCSCs are endowed with the capacity for self-renewal and multi-lineage differentiation, tumorigenicity, and chemotherapy and radiotherapy resistance, features that are responsible for tumor progression, disease recurrence, and metastasis [19–21]. It has been reported that CSC subpopulations are relatively radioresistant compared with non-CSC subpopulations, because of their high DNA repair capability and upregulated survival pathways that protect them from various cellular stresses including radiation. Thus, the development of new potent CSC targeting therapeutics is highly desirable [22–24].

The heavy ion medical accelerator in Chiba (HIMAC) at the National Institute of Radiologic Science (NIRS) has treated more than 9000 patients with various radioresistant tumors, and achieved promising results to date [25–27]. The heavy ion beams have a well-defined range and insignificant scatter in tissues with well-localized energy deposition at the end of the beam path, called the “spread out bragg peak (SOBP)”, a unique physical characteristic of charged particle beams, and release enormous energy at the end of their range. They therefore induce more cell cycle- and oxygenation-independent, irreparable DNA damage and kill more resistant cancer cells than conventional radiation [28, 29]. Recently, a phase I clinical trial of early stage BC treatment with heavy ion radiotherapy was started. However, because of limitations of dose elevation because of side effects on skin, ribs, and lungs after carbon ion radiotherapy, especially for some aggressive subtypes of BC like TNBC, we thought that carbon ion beam combined with chemotherapy may reduce the doses of irradiation but still have some advantage to destroy BC. The combination of chemotherapy with heavy ion radiotherapy may open new perspectives in the fight against this challenging BC subgroup with worse prognosis and still limited therapy options.

Recently, we have reported that BCSCs can be generated by steroid hormones in irradiated breast cell lines [30], and also shown that a carbon ion beam has a marked effect on colon and pancreatic CSCs, which are resistant to photon beams [31, 32]. Considering the fact that cisplatin (CDDP) has been reported to be effective in treating TNBC [33], in the present study, we try to examine the effects of a carbon ion beam alone or in combination with CDDP on putative BCSCs survival, DNA repair, and expression changes of various genes compared to that of X-ray irradiation. To the best of our knowledge, this is the first study to show heavy ion radiation combined with CDDP has an advantage in targeting BCSCs at relatively low doses compared to carbon ion beam alone or conventional X-ray irradiation.

## Materials and methods

### Cell lines and reagents

Human triple negative breast cancer cell lines, MDA-MB-231 and MDA-MB-453 were purchased from American Type Culture Collection (Manassas, VA). Unsorted cells were cultured in Dulbecco's Modified Eagle's medium (DMEM) supplemented with 10 % heat-inactivated fetal bovine serum (Beit-HaEmek, Israel), 100 unit/mL penicillin and 100 μg/mL streptomycin (Invitrogen) at 37 °C with 5 % CO2-in-air. The medium was changed every other day. CSCs and non-CSCs isolated from MDA-MB-231 and MDA-MB-453 cells were cultured with serum-free Essential 8 medium (Life technologies Japan Ltd, Tokyo). CDDP was purchased from Sigma Japan. The CDDP solutions were diluted in PBS immediately before use.

### Animals

NOD/SCID mice (6–8 weeks old, Charles River Laboratories, Yokohama, Japan) were maintained under defined conditions at the NIRS animal facility. The animals were observed at least 24 weeks, and tumorigenicity was determined when tumor nodules were identified on their body surfaces. Tumor formation assay for MDA-MB-231 delivered CD44+/CD24- and CD44-/CD24- cells and MDA-MB-453 delivered ESA+/CD24- and ESA-/CD24+ cells were also performed as described previously [32]. All experiments involving the use of animals were performed in accordance with NIRS institutional animal welfare guidelines.

### Colony and spheroid formation assays

Clonogenic survival assay was performed as described previously [31, 32]. In brief, the appropriate plating density was aimed at producing 20–40 surviving colonies in each T-25 flask. After incubation for 14 days, the colonies were fixed and stained with 0.3 % methylene blue in ethanol, and colonies containing more than 50 cells were counted as survivors. At least three parallel samples were scored in three to five repetitions performed for each type of irradiation. Clonogenicity and/or spheroid formation ability assay for CD44+/ CD24- and CD44-/CD24- cells sorted from MDA-MB-231 cells and ESA-/CD24+ and ESA+/CD24- cells sorted from MDA-MB-453 cells were performed as described previously [31]. The data is presented as the percentage of the wells that contained spheres, and the average size using WinRoof 5.6 software (Mitani Corporation, Tokyo, Japan) after 1-week incubation.

### Irradiation

Cells were irradiated with carbon-ion beams (accelerated by the HIMAC). Briefly, the initial energy of the carbon-ion beams was 290 MeV/n, 50 KeV/μm, center of 6 cm Spread-Out Bragg Peak (SOBP). As a reference, cells were also irradiated with conventional 200 kVp X-ray (TITAN-320, GE Co., USA).

### FACS analysis

FACS analysis for the cells irradiated with X-rays or carbon ion beams was performed with BD FACS Aria (Becton Dickinson, San Jose, CA, USA) as described previously [31]. In brief, the cells were prepared and labeled with conjugated anti-human CD44-PE (Miltenyi Biotec), ESA-APC (Miltenyi Biotec), and CD24-FITC. Isotype matched immunoglobulin served as control. Cells were incubated for 20 min at each step and were washed with 2 % FBS/PBS between steps. The percentage of CD44+, ESA+, and CD24+ present was assessed after correction for the percentage of cells reactive with an isotype control.

### Cell cycle analysis

After harvesting and washing cells with PBS, they were fixed in ice-cold 70 % ethanol **(**ethanol in distilled water) while vortexing, then stained with propidium iodide (1 μg/mL, Sigma) in the presence of RNase A, and then analyzed using a BD FACS Calibur flow cytometer (BD Biosciences). A minimum of 10,000 cells were counted for each sample, and data analysis was performed with CellQuest software.

### PCR Profiler array analysis of various gene expression related to apoptosis, autophagy and DNA repair

The Human Custom RT^2^ Profiler™ PCR Array (CAPH11870A, Qiagen) profiles the expression of 42 genes involved in DNA damage, apoptosis, autophagy, and angiogenesis. RNA was purified using the Qiagen RNAeasy kit, including on-column DNAse treatment to remove genomic DNA. cDNA was prepared with the RT^2^ First Strand Kit (SABiosciences, Frederick, Maryland, USA). A PCR profiler array specific for 48 × 2 OSRGs was performed (RT^2^ SYBR Green/ROX qPCR Master Mix; SABiosciences) in 96-well microtiter plates on an ABI 7300 instrument (Applied Biosystems, California, USA). For data analysis, the ΔΔCt method was applied using the RT^2^ Profiler PCR Array software package and statistical analyses performed (*n* = 3). This package uses ΔΔ C_T_–based fold change calculations and the Student’s *t*-test to calculate two-tail, equal variance p-values. The fold changes were calculated using the equation 2^−ΔΔCt^. If fold change was greater than 1, the result was considered as fold-upregulation. If fold change was less than 1, the negative inverse of the result was considered as fold-downregulation [34].

### γH2AX Immunofluorescence assay

γH2AX Immunofluorescence assay was performed as described previously [32]. In brief, cultured cells grown on plastic chamber slides (Lab-Tek. Nunc, USA) were fixed in 4 % formaldehyde for 15 min at room temperature. Then the cells were permeabilized in 0.2 % Triton X-100 and blocked with 10 % goat serum, then incubated with mouse monoclonal anti-phospho-Histone H2AX(Ser139) (γH2AX) at 37 °C in PBS with 10 % goat serum and washed with PBS. The cells were incubated with the Alexa 488 anti rabbit secondary antibody at 37 °C in PBS with 10 % goat serum and washed in PBS. Cover glasses were mounted in ProLong® Gold antifade reagent with DAPI (Invitrogen). Fluorescence images were captured using an Olympus DP70 fluorescence microscope for analysis. All treatment groups were then assessed for γH2AX foci via sequential imaging through each nucleus. A minimum of 100 cells in each treatment group were counted. Nuclear γH2AX foci size was estimated by WinRoof 5.6 software (Mitani Corporation, Tokyo, Japan) .

### Statistical analysis

One-way analysis of variance (ANOVA) and Bonferroni multiple comparison tests were used when mean differences between the groups were evaluated by StatView software (SAS Institute, Inc., Cary, NC). For all comparisons, *p* values less than 0.05 were defined as significant.

## Results

### *Determination of cancer stem-like cell properties of* CD44+/CD24- *and ESA+/CD24- cells sorted from MDA-MB-231 and MDA-MB-453 cells*

**Fig. 1:**
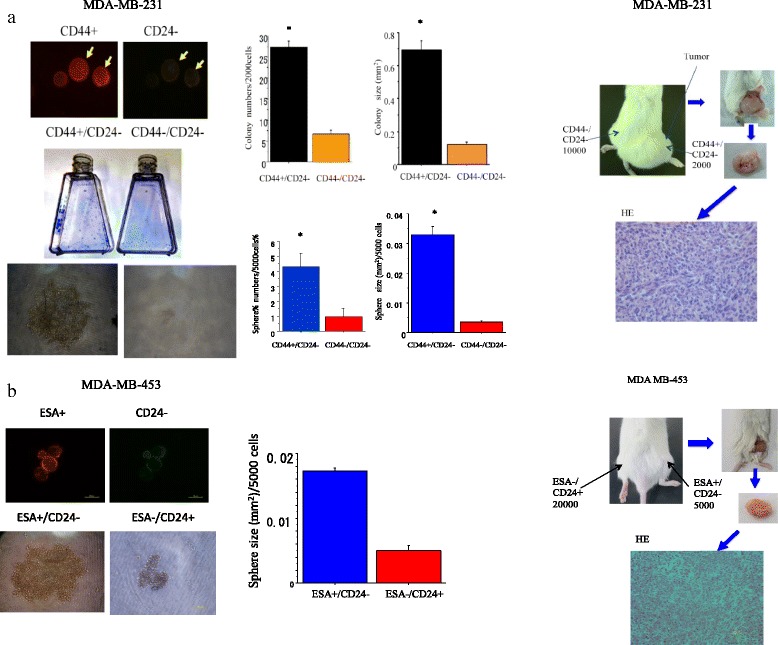
**a**. Colony, spheroid formation and tumorigenicity of cancer stem-like cells (CSCs) (CD44+/CD24-) and non-CSCs (CD44-/CD24-) delivered from MDA-MB-231 cells. The cells were cultured for 1–2 weeks or injected into NOD-SCID mouse right and left thighs for 12–16 weeks for colony, spheroid and tumor formation ability analyses. **b**. Spheroid formation and tumorigenicity of cancer stem-like cells (CSCs) (ESA+/CD24-) and non-CSCs (ESA-/CD24-) delivered from MDA-MB-453 cells. The cells were cultured for 1–2 weeks or injected to NOD-SCID mouse right and left thighs for 12–16 weeks for colony, spheroid and tumor formation ability analyses. CSCs formed tumor HE is displayed. Representative photos of CSCs are also displayed. *, *p* < 0.01, compared to colony or sphere formed from non-CSCs. All experiments were performed in triplicate (*n* = 3)

As shown in Fig. 1, CD44+/CD24- cells had greater colony and sphere formation abilities than CD44+/CD24- cells. When an equal number of 5000 cells were plated in a dish, CD44+/CD24- cells from MDA-MB-231 formed 135 + 6 clones, whereas CD44+/CD24- cells formed only 78 + 2 clones (p < 0.01). These data showed that CD44+/CD24- BC cells had much greater clonal formation capacities than those of CD44-/CD24- cells (Fig. 1a). To investigate the ability to form spheroid bodies, isolated CD44+/CD24- and CD44-/CD24- cells were cultured in 96-well round-bottomed Sumilon celltight spheroid plates (Sumilon, Sumitomo Bakelite Co., Tokyo, Japan). After being in culture for 1 week, the ability of CD44+/CD24- cells to form spheroid bodies was significantly higher both in number and in size than that of CD44-/CD24- (p < 0.01) (Fig. 1a). CD44 was almost undetectable but the ESA was detectable from MDA-MB-453 cells. As shown in Fig. 1b, the spheroid formation ability of ESA+/CD24- is significantly higher than ESA-/CD24+ cells sorted from MDA-MB-453 cells.

To examine in vivo tumorgenicity, various numbers of CD44+/CD24- or CD44-/CD24- cells isolated from MDA-MB-231, and ESA+/CD24- or ESA-/CD24+ isolated from MDA-MB-453 cells were subcutaneously transplanted into the right or left lower thigh of immunodeficient NOD SCID mice. As shown in Fig. 1 and Additional file 1: Table S1, only 5000 cells of CD44+/CD24-**/** cells could form a tumor whereas 1 × 10^4^ CD44-/CD24- or ESA-/CD24+ cells could not, suggesting that CD44+/CD24-and ESA+/CD24- cells have characteristics of cancer stem-like cells.

### *Changes in proportion of* CD44+/CD24- *and ESA+/CD24- cells after carbon-ion beam alone or in combination with CDDP*

**Fig. 2:**
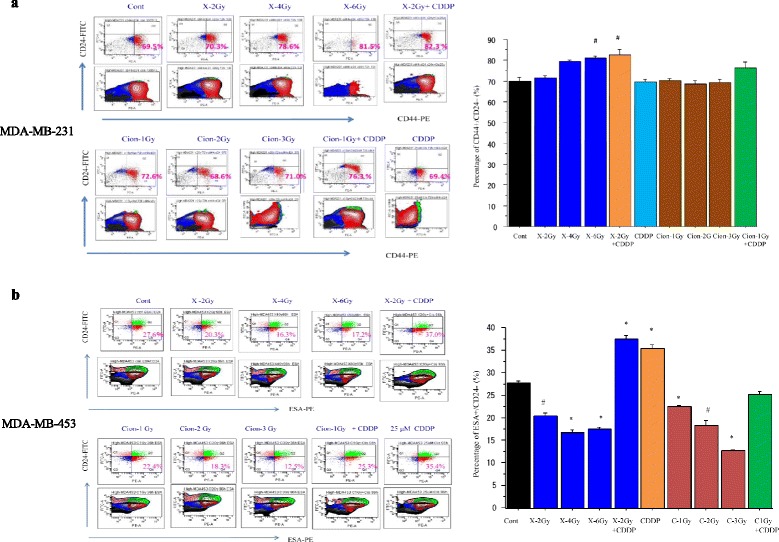
**a**. Percentage changes of CD44+/CD24- cells by FACS analysis 72 h after carbon ion beam or X-ray irradiation alone or in combination with 25 μM of cisplatin (CDDP) in MDA-MB-231 cells. CDDP was added prior to irradiation and treated for 72 h. **b**. Percentage changes of ESA+/CD24- cells by FACS analysis 96 h after carbon ion beam or X-ray irradiation alone or in combination with 25 μM of CDDP in MDA-MB-453 cells. CDDP was added prior to irradiation and treated for 96 h. #, p < 0.05; *, p < 0.01 compared to non-CSCs. All experiments were performed in triplicate (*n* = 3)

The percentage changes of cancer stem like CD44+/CD24- cells in MDA-MB-231 cells and ESA+/CD24- cells in MDA-MB-453 cells 72 h or 96 h after carbon ion beam, X-ray alone or in combination with 25 μM of CDDP were investigated by FACS analysis. As shown in Fig. 2a, the proportion of CD44+/CD24- cells was dose dependently increased after X-ray irradiation, whereas no significant changes by carbon ion beam at which the doses induced equivalent effects by X-ray. The percentage of CD44+/CD24- cells was increased more significantly when X-ray combined with CDDP compared to that of carbon ion beam with CDDP alone. However, the proportion of ESA+/CD24- cells in MDA-MB-453 cells was decreased either by X-ray or carbon ion beam alone, but significantly increased by combination with CDDP or with CDDP alone (Fig. 2b).

### Surviving fraction of unsorted MDA-MB-231 cells and sorted CSCs and non-CSCs after carbon ion beam or X-ray irradiation

**Fig. 3:**
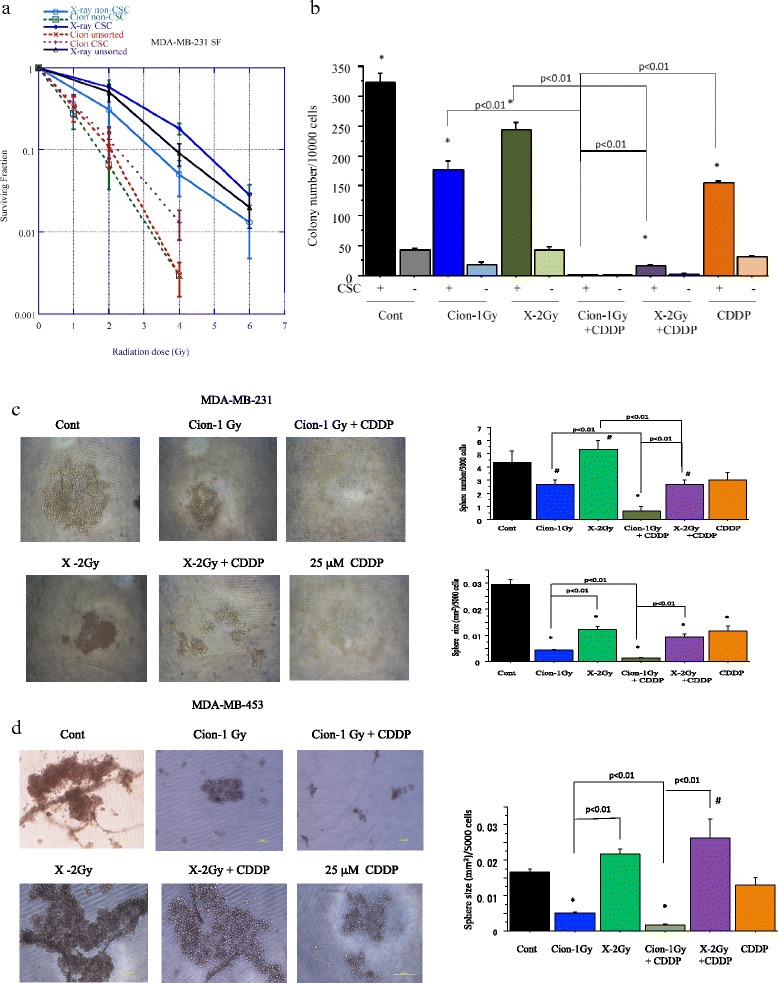
**a**. Surviving fraction of unsorted MDA-MB-231 cells and CSCs (CD44+/CD24-) and non-CSCs (CD44-/CD24-) delivered from MDA-MB-231 cells plated immediately after carbon ion beam or X-ray irradiation. The graphs show the mean and standard error calculated from three independent experiments. All experiments were performed in triplicate (*n* = 3). **b**. Quantification of colony formation of (CD44+/CD24-) and non-CSCs (CD44-/CD24-) after X-ray, a carbon ion beam alone or in combination with 25 μM of CDDP. CDDP was added prior to irradiation and treated for 5 days. *, *p* < 0.01, compared to non-CSCs. Representative photos and quantification of spheroid size formed from MDA-MB-231 delivered CSCs (CD44+/CD24-) (**c**) and MDA-MB-453 delivered CSCs (ESA+/CD24-) (**d**) after X-ray, a carbon ion beam alone or in combination with 25 μM of CDDP. The spheroid formation was observed 7 days after X-ray, a carbon ion beam alone or in combination with CDDP. CDDP was added prior to irradiation and treated for 5 days. The graphs show the mean and standard error calculated from three independent experiments. *, *p* < 0.01, compared to non-CSCs. All experiments were performed in triplicate (*n* = 3)

The MDA-MB-231 cells were irradiated with X-ray or carbon ion beams and their surviving fraction was estimated by colony assay. The surviving fractions for the MDA-MB-231 cells irradiated with X-ray or carbon ion beam decreased exponentially with increasing doses. Based on the survival curve, the D10 (dose required to reduce the surviving fraction to 10 %) was estimated as 3.9 Gy for X-ray and 2.0 Gy for carbon ion beam. Therefore the relative biological effectiveness (RBE) values for SOBP carbon ion beams relative to X-rays at **D10** level is about 1.80 (Fig. 3a).

The surviving fractions for the cancer stem-like CD44+/CD24- and non-cancer stem like CD44-/CD24- cells sorted from MDA-MB-231 irradiated with X-rays and carbon ion beams decreased exponentially with increasing doses, and CD44-/CD24- cells more significantly decreased compared to that of CD44+/CD24- cells after irradiation with either X-rays or carbon ion beams (Fig. 3a). The RBE values calculated at the D10 level for CSCs were calculated to be about 2.14, whereas those for non-CSCs were about 1.78. RBE values for unsorted and sorted CSCs and non-CSCs of carbon ion beams relative to X-rays are summarized in Table 1.

**Table 1:** RBE values at D10 level for unsorted MDA-MB-231 cells and sorted cancer stem-like and non-cancer stem-like cells

Cells	X-ray	C-ion	RBE
MDA-MB-231 unsorted CD44+/CD24-	3.9 ± 0.11 Gy	2.0 ± 0.05 Gy	1.80
	4.52 ± 0.12 Gy	2.12 ± 0.11 Gy	2.14
CD44-/CD24-	3.21 ± 0.11 Gy	1.82 ± 0.06 Gy	1.78

### Colony and/or spheroid formation ability of CD44+/CD24- and ESA+/CD24- cells sorted from MDA-MB-231 and MDA-MB-453 cells after carbon-ion beam or X-ray alone or in combination with CDDP

To examine the effects of cisplatin on radiosensitization to X-rays and carbon ion beams, colony as well as spheroid formation ability of cancer stem-like CD44+/CD24- cells and non-cancer stem-like CD44-/CD24- cells after irradiation with an X-ray or carbon ion beam alone or in combination with CDDP were performed. We found that the number of colonies from both CSCs and non-CSCs was remarkably reduced when carbon ion beam combined with 25 μM of CDDP compared to carbon ion beam alone or X-ray combined with CDDP (Fig. 3b). As shown in Fig. 3c, the number of tumor spheroid formations of cancer stem like CD44+/CD24- cells delivered from MDA-MB-231 cells was significantly reduced after carbon ion beam compared to X-ray irradiation, and it was extremely decreased when the carbon ion beam combined with CDDP. In contrast, there are no spheres formed in non-cancer stem-like CD44-/CD24- cells after X-ray or carbon ion beam, either alone or in combination with CDDP. Spheroid formation ability of ESA+/CD24- cells sorted from MDA-MB-453 after irradiation with an X-ray or carbon ion beam alone or in combination with CDDP was also performed, and showed the carbon ion beam alone but not X-ray significantly inhibited spheroid size and it was remarkably suppressed when carbon ion beam combined with CDDP (Fig. 3d).

### Expression changes of various genes in CSCs after carbon-ion beam alone or in combination with CDDP by RT PCR Array analysis

**Fig. 4:**
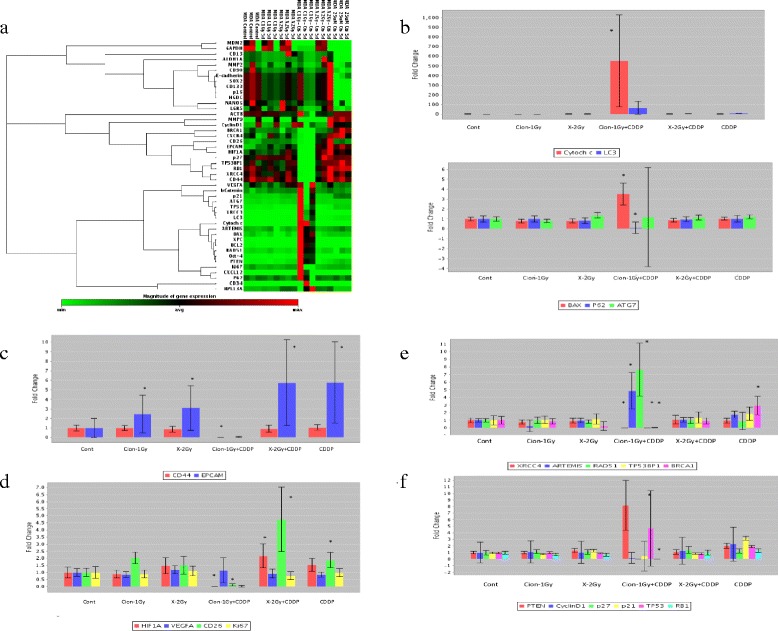
**a**. Clustergram of Custom RT-PCR Array of CSCs delivered from MDA-MB-231 cells 5 days after a carbon ion beam, X-ray alone or in combination with 25 μM of cisplatin (CDDP). CDDP was added prior to irradiation and treated for 5 days. **b**. Expression of apoptosis- and autophagy-related genes after carbon ion beam, X-ray alone or in combination with CDDP in CSCs. **c**. Expression CSC markers after carbon ion beam, X-ray alone or in combination with CDDP in CSCs. **d**. Expression of angiogenesis-, metastasis-related genes after carbon ion beam, X-ray alone or in combination with CDDP in CSCs. **e**. Expression of DNA repair-related genes after carbon ion beam, X-ray alone or in combination with CDDP in CSCs. **f**. Expression of cell cycle-related genes after carbon ion beam, X-ray alone or in combination with CDDP in CSCs. *, *p* < 0.01, compared to control. All experiments were performed in triplicate (*n* = 3)

To quantitatively examine multiple gene expression changes in radioresistant CSCs (CD44+/CD24-) delivered from MDA-MB-231 cells, RT^2^ Profiler PCR Array analysis was performed according to the manufacture’s protocol. A representative clusterhistogram is shown in Fig. 4a. The data shows that treatment with a carbon ion beam combined with constant treatment with 25 μM of CDDP for 5 days significantly increased the expressions of apoptosis-related Cytochrome c, and had a strong tendency to increment Bax (*p* = 0.059) and autophagy-related genes LC3 (*p* = 0.057) compared to carbon ion beam, X-ray, cisplatin alone or X-ray combined with CDDP (Fig. 4b). In addition, expressions of CSC markers, CD44 and ESA were almost completely eliminated by carbon ion beam combined with CDDP, whereas X-ray, carbon ion beam, CDDP alone or X-ray combined with CDDP (Fig. 4c) significantly increased expression of ESA. Besides, expressions of angiogenesis- and metastasis-related genes such as HIF1α and CD26 were remarkably inhibited or lost by carbon ion beam combined with CDDP, whereas cisplatin alone or X-ray combined with CDDP significantly increased expressions of HIF1α and CD26 (Fig. 4d). Interestingly, some DNA repair-related genes such as XPC, Artemis, Rad51, and cell cycle-related gene PTEN were remarkably elevated but some of them, like XRCC4, 53BP1, and BRCA1, p27, and RB1 were significantly reduced by carbon ion beam combined with CDDP compared to carbon ion beam, X-ray, CDDP alone or X-ray combined with CDDP (Fig. 4e, f).

### Cell cycle analyses of MDA-MB-231 and MDA-MB-453 cells after carbon-ion beam alone or in combination with CDDP

**Fig. 5:**
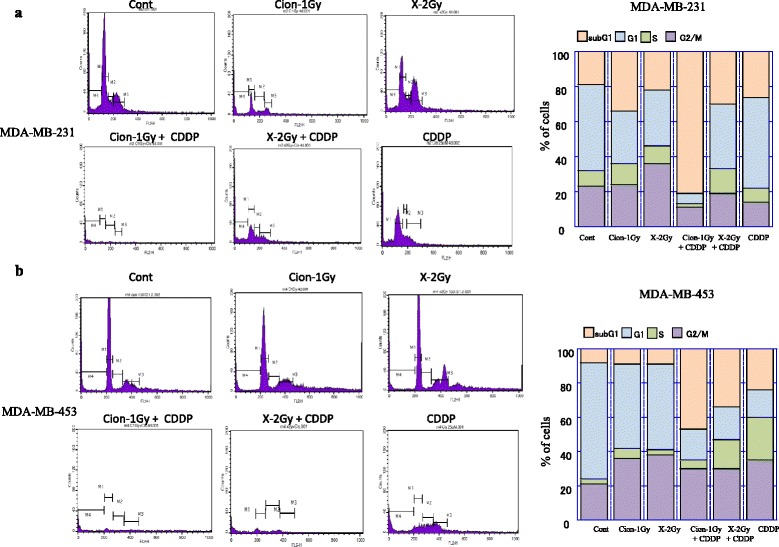
Cell cycle analyses of MDA-MB-231 (**a**) and MDA-MB-453 cells (**b**) 4 days after a carbon ion beam, X-ray alone or in combination with 25 μM of cisplatin (CDDP). CDDP was added prior to irradiation and treated for 4 days, and the cell cycle distribution (sub G1, G1, S and G2/M phase) was measured by flow cytometry. Carbon ion beam combined with CDDP significantly inhibited cell cycle progression (sub-G1 arrest) and induced cell death (apoptosis/necrosis). Three separate experiments were conducted, and representative results are shown. Averages of the three separate experiments are shown in the graph

Cell cycle analyses of MDA-MB-231 and MDA-MB-453 cells 4 days after a carbon ion beam, X-ray alone or in combination with 25 μM of CDDP were performed. CDDP was added prior to irradiation and constantly treated for 4 days, and the cell cycle distribution (sub G1, G1, S and G2/M phase) was measured by FACS Calibur. As shown in Fig. 5, carbon ion beam combined with CDDP more significantly inhibited cell cycle progression (sub-G1arrest) and induced cell death (apoptosis/necrosis) compared to carbon ion beam, X-ray alone or X-ray combined with CDDP both in MDA-MB-231 and MDA-MB-453 cells.

### γH2AX foci formation in CD44+/CD24- and ESA+/CD24- cells after carbon-ion beam alone or in combination with CDDP

**Fig. 6:**
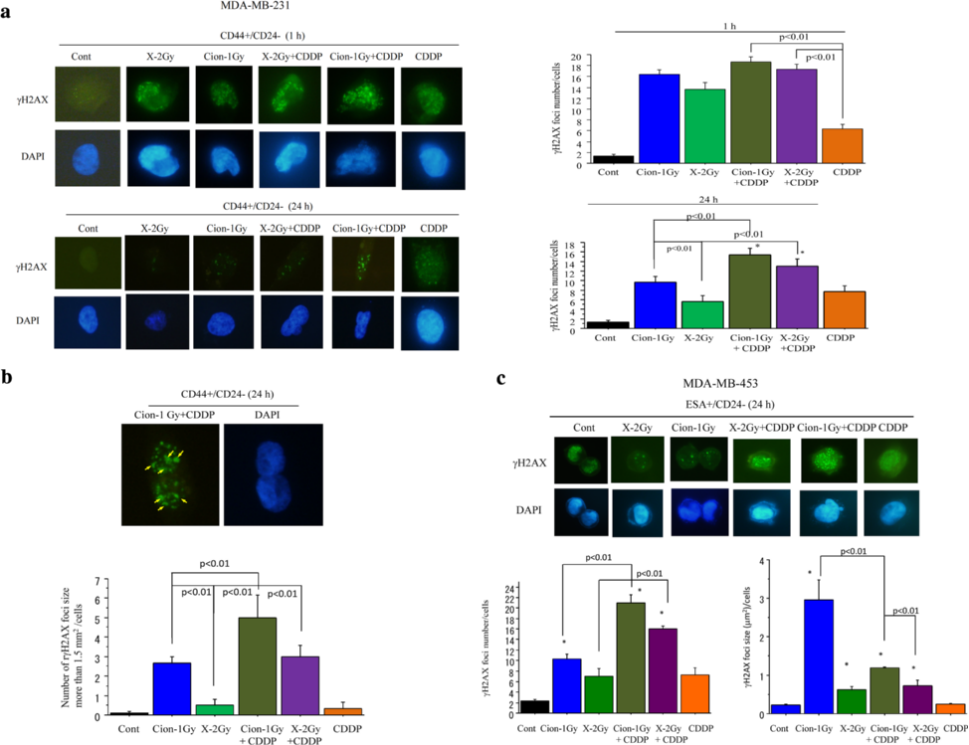
**a**. Quantification and representative photos of nuclear γH2AX foci formation in CSCs (CD44+/CD24-) delivered from MDA-MB-231 cells at 1 h and 24 h after a carbon ion beam, X-ray alone or in combination with 25 μM of cisplatin (CDDP). CDDP was added prior to irradiation and treated for 24 h. Data represent mean ± SD. *p < 0.05 compared to non-CSCs. **b**. Quantification and representative photos of nuclear γH2AX foci larger than 1.5 μm^2^ after 24 h carbon ion beam combined with 25 μM of CDDP in CSCs (CD44+/CD24-) delivered from MDA-MB-231 cells. Data represent mean ± SD. *p < 0.01 compared to CDDP alone treated cells. **c**. Quantification and representative photos of nuclear γH2AX foci formation in CSCs (ESA+/CD24-) delivered from MDA-MB-453 cells at 24 h after a carbon ion beam, X-ray alone or in combination with 25 μM of CDDP. CDDP was added prior to irradiation and treated for 24 h. Data represent mean ± SD. *p < 0.05 compared to non-CSCs. Arrows indicate γH2AX foci larger than 1.5 μm^2^. *p < 0.01 compared to γH2AX foci sizes in X-ray irradiated cells. All experiments were performed in triplicate (*n* = 3)

A high number of γH2AX foci formed at 1 h after a carbon ion beam, X-ray alone, and in combination with 25 μM of CDDP further increased the number of γH2AX foci in CD44+/CD24- cells sorted from MDA-MB-231 (Fig. 5a). However, at 24 h after carbon ion beam irradiation, the induced γH2AX foci level remained significantly higher than that of X-ray irradiated cells with isoeffective dosages, and carbon ion beam in combination with cisplatin remarkably enhanced the number of γH2AX foci compared to carbon ion beam, X-ray, cisplatin alone or X-ray combined with cisplatin (Fig. 5a). Furthermore, not only a great increase in the number but also in the size of foci (clustered DSB) was frequently found in carbon ion beam combined with cisplatin-treated cells compared to carbon ion beam, X-ray, cisplatin alone or X-ray combined with cisplatin-treated cells (Fig. 5b). We also examined the number and size of nuclear γH2AX foci formed in CSCs (ESA+/CD24-) delivered from MDA-MB-453 cells at 24 h after a carbon ion beam, X-ray alone or in combination with 25 μM of CDDP, and found that a much greater number of γH2AX foci were remained after carbon ion beam combined with CDDP, but surprisingly more larger sized γH2AX foci were induced by carbon ion beam alone compared to X-ray alone or in combination with CDDP (Fig. 6c).

## Discussion

In this study, an *in vitro* colony and spheroid formation analysis as well as an in vivo tumorigenicity study showed that CD44+/CD24- cells have a significantly higher possibility compared to CD44-/CD24- cells which sorted from MDA-MB-231 cells, indicating that CD44+/CD24- cells exactly have CSC properties. We also examined and confirmed that ESA+/CD24- cells have CSC properties compared to ESA-/CD24+ which sorted from MDA-MB-453 cells based on its high spheroid formation and in vivo tumor formation ability.

This is in line with previous reports that CD44+/CD24- and/or ESA+ /CD24- cells are BCSC markers [19, 35, 36]. We also investigated the proportion of ALDH*hi*, a typical BCSC marker, is very low in both MDA-MB-231 and MDA-MB-453 cells (Additional file 2: Figure S1), so the CD44+/CD24- and ESA+/CD24- cells were representatively used as CSCs in this study.

We found that the percentages of cancer stem-like CD44+/CD24- cells in MDA-MB-231 cells were dose-dependently increased after 72 h X-ray irradiation, whereas no such clear dose–response was observed after carbon ion beam (Fig. 2). In contrast, the proportion of ESA+/CD24- cells in MDA-MB-453 cells significantly decreased at 96 h after carbon ion beam alone and surprisingly also by X-ray irradiation alone, but significantly increased by X-ray combined with CDDP or CDDP alone. The finding of CD44+/CD24- cells in MDA-MB-231 cells is consistent with our and other previous reports [31, 32, 37], but it is unclear why the proportion of ESA+/CD24- cells in MDA-MB-453 cells was suppressed by X-ray irradiation in this study. In the present study, the *in vitro* relative biological effectiveness (RBE) value calculated by the D10 relative to the X-ray is about -1.75 to 1.85 for the center of SOBP carbon ion beam on MDA-MB-231 cells. RBE values are known to be dependent on linear transfer energy (LET), and our results are consistent with previous reports using carbon ion beams on several human cancer cells, which reported 1.57-2.60 for 50–80 keV/μm-beams [38]. Based on dose–response curves for cell-killing effect on CSCs and non-CSCs after irradiation with either X-rays or carbon ion beams, the CSCs showed resistance to both X-rays and carbon ions compared to non-CSCs. The RBE values calculated at the D10 level for CSCs delivered from MDA-MB-231 were about 2.14, suggesting that the carbon ion beam has more power to destroy CSCs. In contrast, RBE values at the D10 level for non-CSCs delivered from MDA-MB-231 were only 1.78, implying that the difference in killing breast cancer cells between carbon ion beam and X-ray irradiation might mainly result from the strong effects on CSCs (Fig. 3a). Furthermore, the data shows that carbon ion beam combined with CDDP significantly decreased the number of colonies and the size of spheroids formed from MDA-MB-231 and MDA-MB-453 delivered CSCs compared to X-ray, carbon ion beam, CDDP alone or X-ray combined with CDDP, indicating that BCSCs were significantly radiosensitized when carbon ion beam was combined with CDDP (Fig. 3b, c, d).

In general, it has been suggested that CSC subpopulations are relatively radioresistant compared with non-CSC subpopulations, because of enhanced DNA repair capability with an increased ability to activate DNA damage checkpoint responses following radiation (e.g., activation of Chk1 and Chk2 checkpoint kinases), which serves to slow cell cycle progression and permit repair prior to cell division; quiescent cell cycle status (G0), hypoxic environment and upregulated survival pathways that protect from cellular stress [39]. It has been reported that CDDP radiosensitize breast cancer cells are accompanied with apoptosis and autophagy [40, 41]. In the present study, we found that after treatment with carbon ion beam in combination with CDDP for radioresistant CSCs delivered from MDA-MB-231 cells, not only apoptosis-related gene expressions like Cytochrome c but also autophagy-related genes like LC3 showed significant enhancement or a strong tendency to increase compared to that of carbon ion beam, X-ray and CDDP alone or X-ray combined with CDDP suggesting that carbon ion beam combined with CDDP may have more power to induce multiple cell death (Fig. 4a, b). It has been shown that CSCs are closely associated with radioresistance [42, 43]. In this study, carbon ion beam combined with CDDP almost completely inhibited expression of CD44 and ESA. In contrast, carbon ion beam, X-ray, CDDP alone or X-ray combined with CDDP elevated ESA expression, suggesting that a relatively long-term treatment (5 days) by carbon ion beam combined with CDDP may have strong potential to eradicate BCSCs (Fig. 4c). It has been reported that CDDP can induce differentiation of CSC subpopulations within BC cell lines [44]. Thus, we considered that carbon ion beam combined with CDDP make the CSCs more easily killed. In addition, we surprisingly found that expressions of angiogenesis-related gene HIF1α and metastasis-related gene CD26 were significantly suppressed or lost by carbon ion beam combined with CDDP, whereas carbon ion beam alone or X-ray combined with CDDP increased HIF1α, implying that carbon ion beam in combination with CDDP may effectively inhibit tumor angiogenesis and metastasis (Fig. 4d). It has been known that intra-strand lesions, the primary type of DNA damage caused by CDDP are repaired by the nucleotide excision repair (NER) pathway and exquisite sensitivity to CDDP is observed in testicular cancer, which often presents with low expression of NER proteins, such as XPA and ERCC1 [45–47]. Although we did not examine NER genes in this study, we found that carbon ion beam combined with CDDP significantly altered expression of NHEJ-related genes (suppressed XRCC4 but enhanced ARTEMIS) and HR-related genes (suppressed 53BP1 and BRAC1 but enhanced RAD51), whereas CDDP alone treatment increased BRCA1 expression, but carbon ion beam, X-ray alone or X-ray combined with CDDP were not affect those of genes (Fig. 4e). In addition, carbon ion beam combined with CDDP significantly increased expression of PTEN and p53, but inhibited cell cycle related Cyclin D1, p21 and p27, whereas CDDP alone treatment significantly increased p21 expression, in comparison, carbon ion beam, X-ray alone or X-ray combined with CDDP were not affect those of genes (Fig. 4f). These findings suggest that carbon ion beam combined with CDDP greatly disrupts DNA repair and cell cycle regulation of BCSCs. Furthermore, cell cycle analyses of MDA-MB-231 and MDA-MB-453 cells by flow cytometry after a carbon ion beam, X-ray alone or in combination with 25 μM of CDDP showed that carbon ion beam combined with CDDP more significantly inhibited cell cycle progression (sub-G1 arrest) and induced cell death (apoptosis/necrosis) compared to carbon ion beam alone, X-ray alone, CDDP alone or X-ray combined with CDDP (Fig. 5). All together, carbon ion beam in combination with CDDP appeared to show beneficial effects in inducing various gene expressions in the disruption of TNBCSCs at mRNA levels *in vitro*, and further investigation for those of genes at protein levels *in vitro* and in vivo is needed.

In this study, the number of double strand breaks (DSBs) marker γH2AX foci, formed in CSCs was high at 1 h after either carbon ion beam or X-ray irradiation alone, and combination with CDDP further increased their number, suggesting that CDDP has sensitization to both X-ray and carbon ion beams. At 24 h post-irradiation the number and size of γH2AX foci for the carbon ion beam were significantly higher and larger than for X-ray irradiation, revealing the differential repair capacity of the DSBs induced by the high and low LET radiation in CSCs [48–50]. Furthermore, a more larger number of, as well as a larger-sized γH2AX foci were formed with carbon ion beam combined with CDDP compared to X-ray, carbon ion beam alone or X-ray combined with CDDP, suggesting that higher complexity of clustered DSB was induced by carbon ion beam in combination with CDDP (Fig. 6a, b, c). These results reveal the greater complexity of DSBs induced by high LET radiation combined with chemotherapy, which potentially leads to increased mutagenicity and decreased repairability of the damaged site.

Taken together, because the carbon ion beams have a well-defined range with well-localized energy deposition at the end of the beam path, a unique physical characteristic called “SOBP”, and release enormous energy at the end of their range, carbon ion beams therefore induce more cell cycle- and oxygenation-independent, irreparable DNA damage and kill more radioresistant CSCs than photon beams [31, 32], and combination with a DNA-damaging antitumor compound CDDP [51–53] further enhances those of actions based on the present data. This is partially in line with our recent report that carbon ion beams combined with gemcitabine, a nucleoside analogue that causes cytotoxicity by inducing DNA replication blocks, efficiently eliminate pancreatic CSCs [54]. Our results showed in this study are the first to show that predominant effects of carbon ion beam in combination with CDDP on TNBC cell killing mainly result from efficient eradication of CSCs rather than non-CSCs.

## Conclusions

In summary, the carbon ion beam combined with CDDP has promising advantages for targeting putative BCSCs because of complex DNA damage, increased apoptosis, autophagy, and subsequent cell death at relatively low doses compared to a carbon ion beam alone. All together, our findings show the potential benefits of a carbon ion beam in combination with chemotherapy to target TNBCSCs.

## Additional files

Additional file 1: Table S1. In vivo limiting dilution assays of sorted MDA-MB-231 and MDA-MB-453 breast cancer cells using surface markers (number of tumors formed/number of injections). (DOCX 16 kb) Additional file 2: Figure S1. (PPTX 204 kb)

## Abbreviations

TNBC: Triple-negative breast cancer
HER2: Human epidermal growth factor receptor-2
ER: Estrogen receptor
PR: Progesterone receptor
CSC: Cancer stem-like cell
HIMAC: Heavy ion medical accelerator in Chiba
CDDP: Cisplatin
DMEM: Dulbecco's modified eagle's medium
ANOVA: Analysis of variance
SOBP: Spread-out bragg peak
LET: Linear transfer energy
RBE: Relative biological effectiveness
HIF: Hypoxia inducible factor
NER: Nucleotide excision repair
NHEJ: Non-homologous end joining
HR: Homologous recombination
