# Supplementary material for: Carbon ion beam combined with cisplatin effectively disrupts triple negative breast cancer stem-like cells in vitro
Source: Mol Cancer. 2015 Sep 4;14:166. doi: 10.1186/s12943-015-0429-7 (PMC4560051; doi:10.1186/s12943-015-0429-7)
Supplement: Additional file 1: Table S1. — In vivo limiting dilution assays of sorted MDA-MB-231 and MDA-MB-453 breast cancer cells using surface markers (number of tumors formed/number of injections). (DOCX 16 kb) [file 12943_2015_429_MOESM1_ESM.docx]

**Suppl Table 1.** In vivo limiting dilution assays of sorted MDA-MB-231 and MDA-MB-453 breast cancer cells using surface markers (number of tumors formed/number of injections)

| Groups | 2 x 10^4^ | 1 x 10^4^ | 5 x 10^3^ | 2 x 10^3^ |
| --- | --- | --- | --- | --- |
| MDA-MB-231  Unsorted | 4/5 | 1/5 | 0/6 |  |
| CD44+/CD24- |  | 5/5 | 3/5 | 1/5 |
| CD44-/CD24- |  | 1/6 | 0/5 |  |
| MDA-MB-453  Unsorted | 4/5 | 3/5 | 0/5 |  |
| ESA+/CD24- |  | 4/5 | 2/5 | 1/5 |
| ESA-/CD24- |  | 0/5 | 0/5 |  |
| *p* |  | <0.01 | <0.01 |  |

*p*<0.01 compared with results from marker-negative cells.
