# Supplementary figures and images for: Carbon ion beam combined with cisplatin effectively disrupts triple negative breast cancer stem-like cells in vitro
Source: Mol Cancer. 2015 Sep 4;14:166. doi: 10.1186/s12943-015-0429-7 (PMC4560051; doi:10.1186/s12943-015-0429-7)

## Slide 1
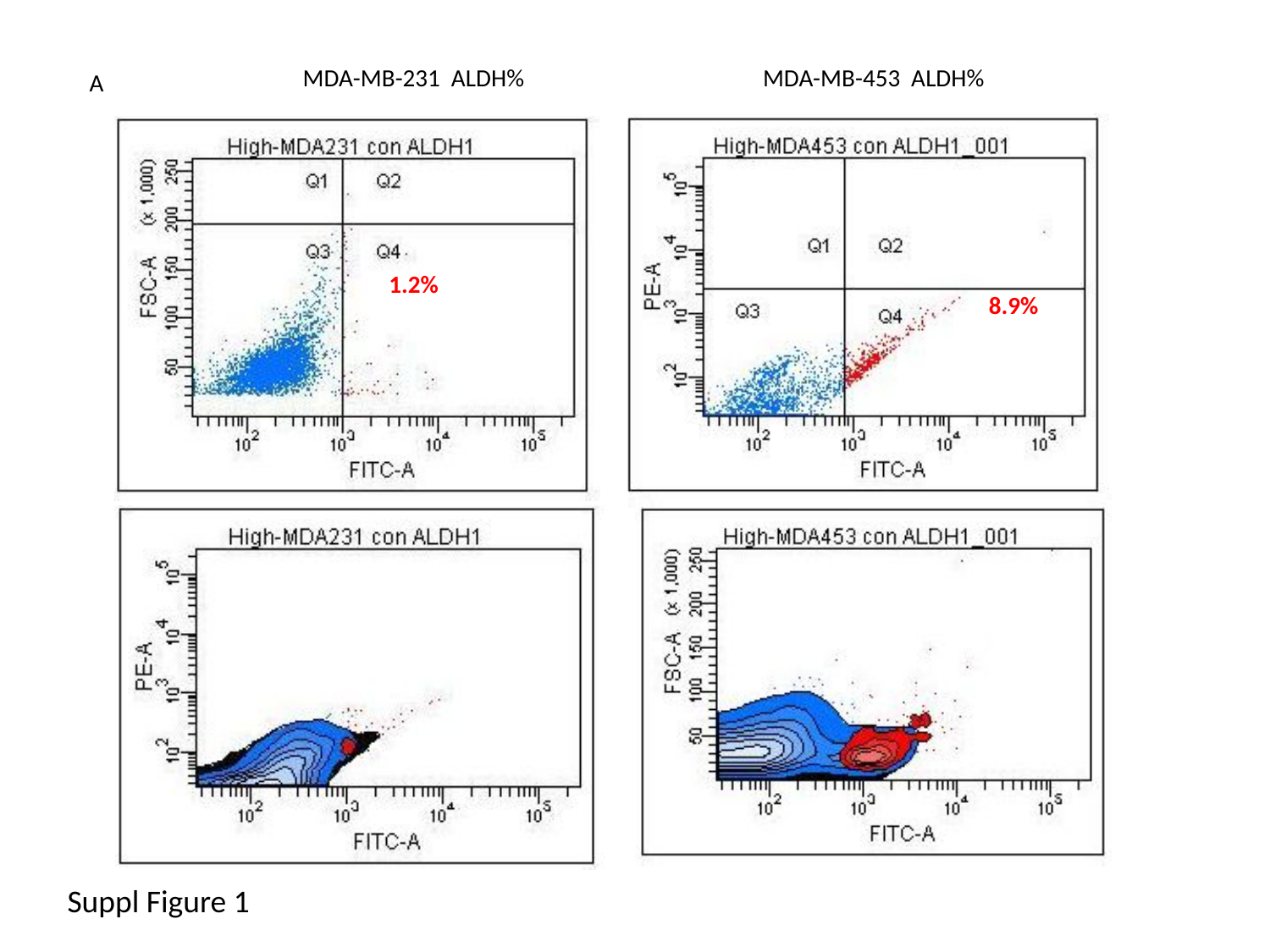

MDA-MB-231 ALDH%
MDA-MB-453 ALDH%
A
1.2%
8.9%
Suppl Figure 1

Supplement: Additional file 2: Figure S1. — (PPTX 204 kb) [file 12943_2015_429_MOESM2_ESM.pptx]
